# Population Pharmacokinetic Modelling of the Complex Release Kinetics of Octreotide LAR: Defining Sub-Populations by Cluster Analysis

**DOI:** 10.3390/pharmaceutics13101578

**Published:** 2021-09-28

**Authors:** Iasonas Kapralos, Aristides Dokoumetzidis

**Affiliations:** 1 Laboratory of Biopharmaceutics-Pharmacokinetics, Department of Pharmacy, National and Kapodistrian University of Athens, 15771 Athens, Greece; iasonaskap@gmail.com; 2 Athena Research and Innovation Center in Information, Communication and Knowledge Technologies, 15125 Athens, Greece

**Keywords:** population pharmacokinetics, octreotide, long acting injectables, machine learning

## Abstract

The aim of the study is to develop a population pharmacokinetic (PPK) model, of Octreotide long acting repeatable (LAR) formulation in healthy volunteers, which describes the highly variable, multiple peak absorption pattern of the pharmacokinetics of the drug, in individual and population levels. An empirical absorption model, coupled with a one-compartment distribution model with linear elimination was found to describe the data well. Absorption was modelled as a weighted sum of a first order and three transit compartment absorption processes, with delays and appropriately constrained model parameters. Identifiability analysis verified that all twelve parameters of the structural model are identifiable. A machine learning method, i.e., cluster analysis, was performed as pre-processing of the PK profiles, to define subpopulations, before PPK modelling. It revealed that 13% of the patients deviated considerably from the typical absorption pattern and allowed better characterization of the observed heterogeneity and variability of the study, while the approach may have wider applicability in building PPK models. The final model was evaluated by goodness of fit plots, Visual Predictive Check plots and bootstrap. The present model is the first to describe the multiple-peak absorption pattern observed after octreotide LAR administration and may be useful to provide insights and validate hypotheses regarding release from PLGA-based formulations.

## 1. Introduction

Octreotide, a biologically stable somatostatin analog, retains its central role in the therapeutics of acromegaly and gastro-entero and pancreatic neuroendocrine tumours (GEP-NETs). Recent clinical trials have widened the perspective of the clinical use of this drug, not only for the management of hormonal hypersecretion, but as an antiproliferative agent, alone or in combination with other drugs. A promising efficacy has been demonstrated with a statistically significant prolongation of time to progression/progression-free survival (TTP/PFS) [1]. The introduction of a long acting repeatable (LAR) formulation of octreotide 25 years ago offered distinctive benefits to patients regarding quality of life and compliance, allowing a single, once per month intramuscular administration.

Octreotide is slowly released from the poly-(lactic-co-glycolic acid) (PLGA) microparticles in which it is encapsulated. It has been confirmed that disposition and elimination occur in different, much faster time scales, suggesting that drug release is the limiting step and pharmacokinetics of octreotide LAR is driven by the mechanisms of delivery from the depot formed by the PLGA vehicle to the systemic circulation. A study of octreotide LAR pharmacokinetics in rats showed that release consists of three phases: an initial burst and two delayed phases, which have been empirically modelled [2]. Drug release from the microsphere depot to the muscle was modelled as the convolution of three processes. The first relates to the rapid release of the drug on or close to the surface of microparticles as water diffuses to the depot, the second phase is driven by diffusion of the drug from the polymeric matrix. Polymeric erosion defines the later phase of delayed release.

The release of drugs from the PLGA microparticle system appears to be a complex process. The interplay between the drug, the formulation and the host, determines the rate of drug delivery. Several factors, including particle size, agglomeration, pore formation and closing, local immune response seem to play an important role, but their effects and dynamics are not yet thoroughly described [3]. It is deemed that a deeper understanding is mandatory for the future development of sustained release formulations based on PLGA, and generic formulations of brand-name drugs.

Population pharmacokinetics (PPK) of octreotide LAR has been previously modelled by the innovator consisting of an initial burst, followed by a zero-order, slow-release phase of the drug, resulting in a plateau [4]. To our view, an appropriate level of granularity in the characterization of the individual pharmacokinetic curves, with respect to the magnitude and the shape of exposure, is needed to provide insights for the mechanistic understanding and evaluate hypotheses regarding drug release from PLGA depot systems. In this study, we present a PPK model of octreotide LAR from a densely sampled PK phase I study in healthy volunteers, which characterizes in detail the complex absorption pattern observed and is capable to simulate realistic, individual subject predictions to generate realistic in silico clinical studies. Furthermore, by tackling the mathematical modelling of the erratic complex absorption patterns, we present, a workflow utilizing machine learning approaches, i.e., clustering, for pre-processing of the raw data in order to optimally characterize the observed heterogeneity and variability of the study.

## 2. Materials and Methods

### 2.1. PK Data

The population PK modelling was performed using data from 118 healthy volunteers, following a single 30 mg intramuscular injection of Sandostatin^®^ LAR Depot (octreotide acetate for injectable suspension, Novartis Pharmaceuticals UK Limited, London, UK), under fasting conditions, as part of a phase 1, single dose PK study. A single dose of deep intramuscular injection was given on Day 0. A pre-dose serum sample was collected on Day 0 and 36 more samples were collected at the following times after administration: 0.5, 1, 1.5, 2, 3, 4, 6, 10, 24, 48, 72, 96, 144, 192, 240, 288, 336, 384, 432, 480, 528, 576, 624, 672, 720, 768, 816, 864, 912, 1008, 1176, 1344, 1512, 1680, 1848 and 2088 h. Two subjects were removed according to the clinical protocol and a dataset was constructed including patients from the reference arm for the population PK analysis purpose. Demographic data, comprising body weight, height, BMI, age, gender and ethnicity were also provided. The study was conducted according to the guidelines of the Declaration of Helsinki and approved by the Jordan Food and Drug Administration (IRB#: TRI-80818). The bioanalysis was carried out by a validated LCMSMS method by Triumpharma CRO (Amman, Jordan). Briefly, the method used a Triple Quad LCMSMS instrument from SCIEX (Framingham, MA, USA) and a ZORBAX SB-C8 column from Agilent Technologies (Santa Clara, CA, USA), with length 100 mm, inner diameter 4.6 mm and particle size 3.5 µm mL, using Octreotide-D8 as internal standard. Linearity was established by preparing an eight-point standard calibration curve in K3EDTA human plasma, covering the Octreotide concentration range 8.835 pg/mL to 4010.010 pg/mL.

### 2.2. Data Analysis

#### 2.2.1. Clustering

Exploratory analysis and visual inspection of the individual pharmacokinetic curves revealed the presence of two distinctive sub-populations. We applied a clustering method implemented in R with kmlShape package to identify patterns in the data. This method is based on the k-means clustering method, but also takes into account the shapes of the curves, rather than only the classical k-means distance. Namely, the “generalized distance of Fréchet” is used, which was introduced by Genolini et al. [5], which is both a generalization of “shape-respecting distance” and classical distance. This method has been developed particularly for the analysis of longitudinal data, as pharmacokinetic data are, because modest variations on delays may be of limited importance, and yet account to large distances according to the classical k-means method. In order to specify the “importance” of the horizontal and the vertical distance, we examined the performance of the method for different values of lambda (λ), the scale parameter of time. The choice of λ affects the results and the capability of the algorithm to converge. It specifies the relative weight of the distance between two curves according to the x-axis and the y-axis. If the x-axis and y-axis had the same scale, λ = 0.1 would give ten times more weight to a vertical offset than to a horizontal offset, for λ = 1 the horizontal and the vertical offsets would have the same importance and for λ = +∞, the horizontal offsets become very expensive and the Fréchet distance converges to the classical maximum distance. The number of clusters was also a user-defined option. The PK data were normalized with the average concentration observed per individual to denote the fraction of the total exposure observed per second. Relative concentrations range from zero to approximately 8, while the independent variable of time ranges from zero to approx. 2000. A choice of lambda, λ = 4 × 10^3^, would roughly assign the same importance to the horizontal (time) offsets and the vertical offsets. This approach allowed the identification of different patterns in release kinetics, without the influence of apparent clearance and, consequently, total exposure.

#### 2.2.2. Population Pharmacokinetic Modeling

Population pharmacokinetics (PK) modelling was performed with the nonlinear mixed-effects modelling method, implemented in NONMEM Version 7.4 (ICON Dublin, Ireland) [6]. Pharmacokinetic analysis included the representation of the dynamical system describing the nonlinear concentration-time course as a system of ordinary differential equations and the selection of the integrated ODE solver with ADVAN13 subroutine in NONMEM. The variability component, comprising the inter-individual variability of the model parameters and the residual unexplained variability, was coded with “MU-referencing”. Fixed-effects (THETAs) and random-effects (Ω matrix) parameters were estimated with a sequence of commands in the NONMEM control file ordering three sequential estimation methods [7]. The first order conditional estimation with interaction (FOCE-I) method seemed to be more stable to initial estimates perturbation to provide better and more stable estimates of THETAs. The stochastic approximation expectation maximization (SAEM) method succeeded to provide precise estimates of the Ω matrix, given the relatively many parameters and the complexity of the model. Finally, an importance sampling (IMP) stage was employed to obtain the appropriate objective function values and to estimate standard errors. [8] The modelling workflow and evaluation was performed through the graphical interface provided by Pirana Version 2.9.9, goodness-of-fit graphics, and visual predictive checks (VPC) were produced in the R packages xpose and xpose4, while nonparametric bootstraps in Perl-speaks-NONMEM (PsN) program were used to estimate the confidence intervals of the model parameters [9,10].

#### 2.2.3. Structural PK Model

**Figure 1:**
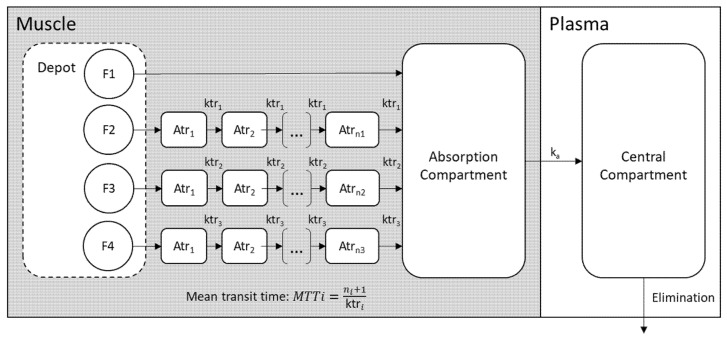
Graphical representation of the pharmacokinetic model which describes octreotide LAR pharmacokinetics. The drug is released from the depot to the muscle through four empirical processes with different kinetic characteristics and is then absorbed to the systematic circulation.

Our data support that octreotide LAR follows a variable, complex, multi-phase pattern. The individual PK curves were visually observed and a typical pattern, comprising the rapid initial burst, followed by up to three release phases with different delays, resulting to four peaks, was identified. The structural model consists of a depot and a central compartment with first-order elimination kinetics (Figure 1). As presented in previous studies, disposition time-scale of hours is much smaller than the release time-scale, which is weeks, so that any additional disposition compartment can be considered to be in equilibrium. The initial burst phase was modelled as a first order process, defined by the absorption rate constant ka. Three parallel delayed processes, using the analytical solution of the transit compartment model developed by Savic et al., were employed to describe the three-phase absorption delays [11]. The input rate in the depot compartment was modelled as a weighted sum of the transit model functions:

(1) $$\frac{dA_{depot}}{dt}=DOSE\times \sum_{j=1}^{3}f_{j}\times TRANSIT_{j}-k_{a}\times A_{a},$$

where *f_j_* stands for the fraction of the dose delivered by the transit process *j* and *TRANSIT_j_* is the function of the *j_ith_* rate of input component, as the following:

(2) $$TRANSIT_{j}=k_{tr_{j}}\times \frac{{\left(k_{tr_{j}}\times t\right)}^{n_{j}}\times e^{-k_{tr_{j}}\times t}}{\sqrt{2\pi \;}\times n_{j}{}^{n_{j}+0.5}\times e^{-n_{j}}}$$

The parameterization, which introduces the mean transit time (*MTT*) instead of single transfer rate constant (*ktr*), was selected. *MTT* represents the average time spent for the drug to reach the absorption compartment, thus it provides a better intuition on release properties. This parameterization also allows the parameters (*MTT_j_*), which correspond to the three parallel transit processes, to be put in sequential order, as following:

(3) $$MTT_{j}=MTT_{j-1}+\theta_{j}\times e^{\eta_{j,i}}$$

Furthermore, an identifiability analysis was performed, applying the method and the software for identifiability analysis popt_i in MATLAB, developed by Shivva et al. [12].

#### 2.2.4. Variability Model

Taking into account the fact that the first delayed release phase, resulting in a local Cmax at approximately 100 h after dose, is present only to a fraction of individuals, *MTT*_1_ was constrained to 300 h, by applying a logit-normal generalization, where the logit term is constraint between 0 and 1:

(4) $${MTT}_{1}=300*\frac{e^{y}}{e^{y}+1},\;y\sim \;N\left(\theta_{1},\;\omega^{2}\right)$$

Fraction parameters *f_j_* indicate the fraction of the bioavailable dose that is released through a process with defined delay and shape. One should not confuse it with F, the absolute bioavailability parameter, which is not identifiable. Thus, the apparent clearance CL/F and the apparent volume of distribution V/F are estimated, and these apparent values are implied everywhere in the text. Therefore, the fraction parameters (*f_j_*) have individual values between 0 and 1, with sums adding up to 1. The logistic-normal transformation described in the article of Tsamandouras et al. [13] was applied to constrain the parameters to the above conditions, so that:

(5) $$f_{1,i}=\frac{e^{u_{1}}}{e^{u_{1}}+e^{u_{2}}+e^{u_{3}}+1},\;where\;u_{j}\;\sim \;N\left(\theta_{j},\;\omega_{j}^{2}\right)$$

Inter-individual variability (IIV) of the remaining PK model parameters was assumed to be log-normally distributed with the following expression for the individual parameter *θ_i_*:

(6) $$\theta_{i}=\theta_{pop}\times e^{\eta_{i}}$$

where *θ_pop_* is the population mean parameter value and *η_i_* is the normally distributed deviation with zero mean and *ω*^2^ variance. IIV was reported as a CV (%) in the original scale, using the equation CV (%) = √(ω^2) × 100%. The variance-covariance matrix Ω was estimated, including the diagonal and the non-diagonal terms, in order to identify correlations in random effects, in the key-models for model-building and the final model. The additive, proportional and combined error model, were tested to describe the residual variability.

Covariate analysis was performed according to the likelihood ratio test (LRT) for significance level of α = 0.01, corresponding to a 6.63 drop in the objective function value. Due to long NONMEM runs, testing all the combinations of parameters and covariates was not feasible. Therefore, only the effect of demographic characteristics on disposition parameters was evaluated, and covariate model building was prioritized by the visual inspection of the post hoc individual parameter estimates vs covariates. The results of the cluster analysis were included in the NONMEM dataset and were handled as a categorical covariate. The covariate model was coded as following:

(7) $$Parameter_{pop}=\theta_{1}+\left(cluster-1\right)\times \theta_{2},\;\mathrm{where}\;\mathrm{cluster}=1\;\mathrm{or}\;2$$

#### 2.2.5. Model Evaluation

The assessment of model adequacy was based on the following criteria: successful minimization, ΔOFV, precision of parameter estimates, successful simulation step, visual inspection of goodness-of-fit plots and visual predictive checks (VPC). To avoid local minima, two or more sets of initial estimates were tested and the model parameters estimated were deemed stable if both runs resulted in similar estimates.

Both prediction-based and simulation-based graphic methods were used for model evaluation at each stage of the modelling-building procedure and for the qualification of the final model. The following goodness-of-fit plots were visually evaluated: Observations vs Individual or Population Predictions (IPRED and PRED), Conditional Weighted Residuals (CWRES) vs. Time or PRED, Observations and IPRED vs Time in the individual level. The violation of model assumptions was assessed through the graphical inspection of ETAs and residuals distributions and q-q plots [14].

After each successful NONMEM run under the model estimates, 1000 datasets were simulated and statistics were computed and compared graphically with the generation of a visual predictive check (VPC), with xpose package in R. The 80%, 90% and 95% prediction intervals and the median of the observations were compared one after another with the corresponding 95% confidence intervals of the simulated data, with the purpose of detecting structural and variability model misspecifications. Non-compartmental analysis (NCA) of both the observed and the simulated PK data was performed by using the NonCompart package in R with the “linear-up log-down” method and VPCs of the PK metrics, AUC (0–28 d), AUC (28–56 d), AUC (0–t), AUC (0–24 h) and Cmax, were produced [15].

The uncertainty of parameter estimation for the final model was evaluated with the 95% confidence interval obtained by the results of a nonparametric bootstrap run with 200 resampled datasets, as implemented in PsN [11]. The resampling procedure was conducted using the stratification option on the cluster variable to handle the proportion of clusters in each resampled dataset.

Furthermore, the robustness of the analysis of the entire workflow comprising the pre-processing step of clustering and the NONMEM run was evaluated by using the bootstrap method. For each one of 200 resampled datasets, two steps were sequentially performed, i.e., the clustering and the model fitting step, using a semi-automated procedure coded in R and using PsN.

## 3. Results

The final dataset used for analysis consisted of 3936 PK observations from 118 individuals, who received a single intramuscular dose of octreotide LAR. Gender and ethnicity data were excluded from the final dataset, because the cohort consisted solely of Caucasian males. One subject (ID #37) was dropped out as an outlier. A summary of demographic characteristics is provided in Table 1.

### 3.1. Clustering

**Figure 2:**
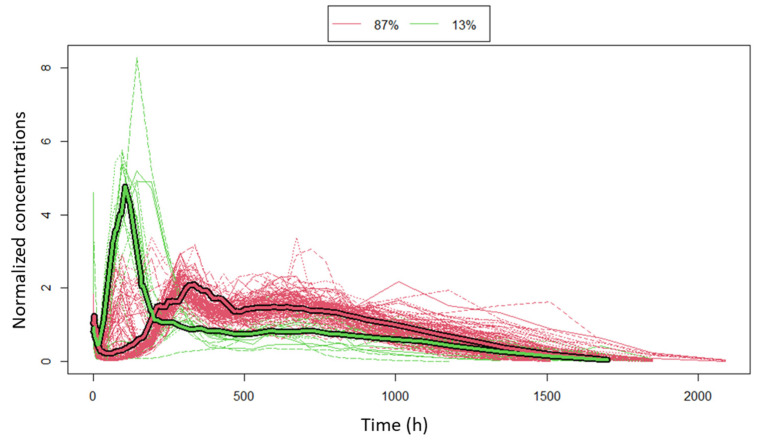
Two sub-populations were identified by the cluster analysis. The clusters are depicted with the different colour and the “mean typical profiles” are drawn with the bold line. Concentrations were normalized with the average concentration per subject to return the shape of exposure, therefore normalized concentrations on the y-axis are unitless.

The PK profiles of the 118 subjects were imported in R and the kmlShape package of R was run. Different values of lambda (λ), the scale factor of time, were employed and the relevance of the results was examined from a pharmacokinetic perspective. The “final” value chosen was λ = 0.001, which showed stability regardless the inclusion or exclusion of a small number of subjects and led to successful algorithm convergence. This value roughly assigns four times more weight to a vertical offset, the space of concentrations, than to a horizontal time offset. The cluster analysis allowed the identification of two different patterns in PK data. We ran the algorithm for a larger number of clusters, but all the individuals were consistently assigned to two clusters. Two typical concentration-time profiles were recognized, as shown in Figure 2. The first one defines cluster 1, consisting of the 87% of the subjects, and presents the aforementioned typical multi-phase pattern of the initial burst and up to three delayed peaks. The 13% of the subjects-cluster 2-were characterized by an early extended phase of absorption, followed by a slow delayed release phase, which corresponds to a small part of the total exposure. Non-compartmental analysis showed that the second cluster has much larger average values of area under the concentration-time curve (AUC), 1.3 × 10^6^ pg × h/mL vs. 0.94 × 10^6^ pg × h/mL, and maximum concentration Cmax, 5034.8 vs. 1433.3 pg/mL, so modelling with respect to this sub-population is important to appropriately predict these measures (Table 1). 

The clustering algorithm involves a stochastic component i.e., the initialization step defines k individuals (k is the user-defined number of clusters) to be chosen from the data. Thus, every re-run of the algorithm or changes in the dataset may lead to slightly different results. To evaluate our analysis, we ran the clustering algorithm for the 200 bootstrap-generated resampled datasets. The occurrence probability of the 12 out of 15 subjects of cluster 2 to be assigned to this cluster was over 93%, while for the other three subjects was over 50%. The dataset consisting of the subjects who received the test product was used for external evaluation of the clustering and similarly, two groups of 13% (16/119) and 87% (103/119) of the subjects with similar “typical PK profiles” were identified.

### 3.2. Population PK Model

The base model consisted of the one-compartment disposition model with linear elimination, coupled with the empirical absorption model described above. Estimates of the model parameters and their relative standard errors (RSE) are provided in Table S1. The full variance-covariance matrix, including the non-diagonal terms, was estimated and evaluated in the course of model development. The covariance terms between the fraction IIV parameters and the mean transit time IIV parameters were considered significant and improved the overall fit, therefore they were kept in the base model. IIV was estimated for all PK parameters with good precision, except for parameter ka, which was not estimated. The PPK model we applied was found to be structurally identifiable, while the parameter space in which it is internally identifiable was explored. The sequential order of MTT was deemed to be crucial to avoid a flip-flop phenomenon regarding the release phases definition. The multi-variate logistic normal distribution of the fraction parameters was also important from a identifiability analysis point of view.

**Figure 3:**
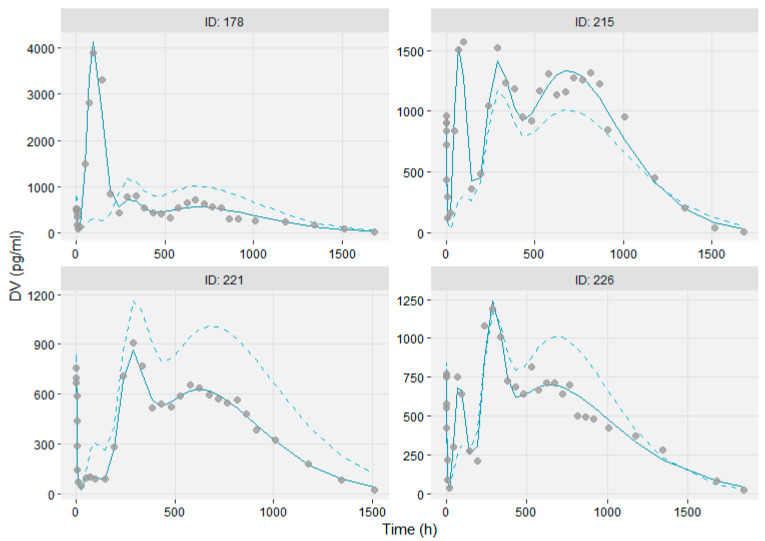
Individual plots of Observations vs PRED and IPRED showing the flexibility of the PPK model to predict for four representative subjects. Solid line and dashed line indicate IPRED and PRED, respectively.

**Figure 4:**
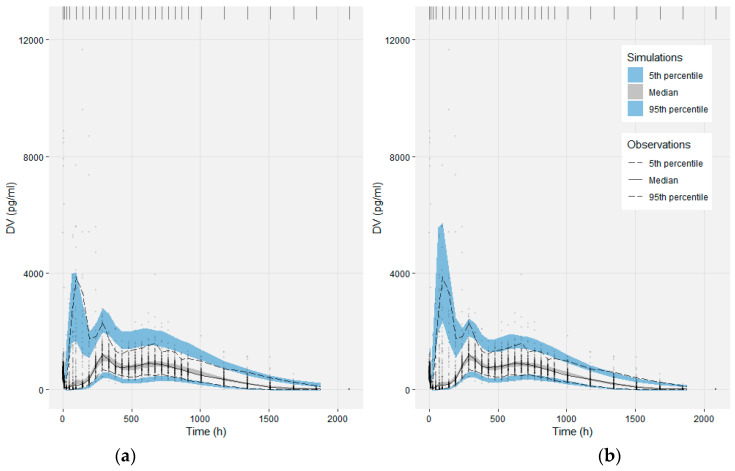
Visual Predictive Checks of the base model (**a**) and the final model (**b**). The median, 5th and 95th percentiles of the observations (lines) are compared with the corresponding 95% confidence intervals of the 1000 simulated datasets (shaded areas).

The base model sufficiently described the complex and highly variable individual PK profiles, as seen in the individual Observations vs IPRED plots of Figure 3. A minor model misspecification was evident in the Observations vs IPRED plot in the logarithmic scale (Figure S1), as the base model underpredicts the low concentrations observed at the terminal slope, approx. two months (over 1850 h) after drug administration. The empirical drug release model was not capable of describing all the deviations observed in the population. The VPC in Figure 4a suggests that the base model describes well the median, and at a satisfactory level the 5th and 95th percentiles of the observed data, taking into account the large variance on data.

ETA-shrinkage of the IIV for all model parameters was less than 30%; with the exception of the IIV of N2, the number of the transit compartments associated with the second peak, which was 30.9%, indicating the reliable Empirical Bayesian Estimates (EBE) of the model parameters and IPRED estimates. A notable observation was that the EBEs of parameter YF2, the normally distributed parameter associated with F2, followed a bimodal distribution, violating the normality assumption of the variability model for this parameter. Intuitively, the fraction parameter F2 is the fraction of dose attributed to the first delayed transit process, resulting in the second peak, and ranges from the absence of a second peak to an overall maximum. This finding supports our hypothesis, that two distinctive PK profiles are present after the administration of octreotide LAR. The EBEs of the remaining parameters and the residuals in general satisfy the symmetry assumptions.

### 3.3. Modeling the Sub-Populations of Cluster Analysis

**Figure 5:**
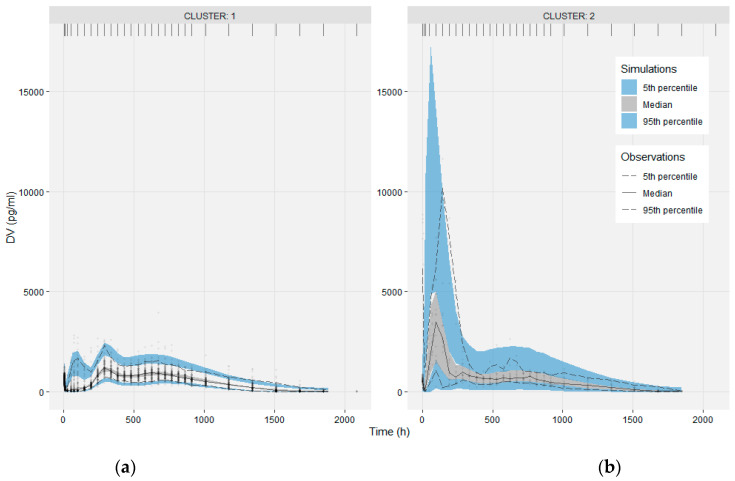
Visual Predictive Checks of the final model, stratified on cluster, (**a**) for cluster 1 and (**b**) for cluster 2.

Cluster results were handled as a binary categorical covariate and its effect on model parameters was evaluated. Covariate model building was guided by the visual inspection of the base model’s EBEs vs clusters. A correlation between clusters and the model parameters F2, F3, MTT1, N1 and CL was observed, that set the prioritization in the evaluation of the cluster covariate effect. The final decision was based on the criteria discussed in the methods section, considered as a whole. The final model incorporated the cluster effect on the model parameters of F2, F3 and CL. This resulted in a drop in the objective function value, ΔOFV = −115.158, which corresponds to a statistically significant result, according to the LRT, for a confidence level of 0.01 and three degrees of freedom, the three parameters of the covariate effect. The relative standard errors associated with the parameters of the covariate effect were low at approximately 30%. The inclusion of the covariate effect decreased the IIV of the corresponding parameters, while the ETA-shrinkage remained unchanged. Cluster effect moderately succeeded to describe the bimodality observed in the distribution of the base model F2 EBEs. The performance of the final model was evaluated, and the goodness-of-fit plots are provided in Figure S2. The VPC of Figure 5 for the final model, stratified on cluster, shows the better overall performance of the model to describe the observed data of the two sub-populations. Parameter estimates, along with the 90% bootstrap confidence intervals, are presented in Table 2, supporting the stability and robustness of the estimation.

The robustness of the analysis comprising the pre-processing step of clustering was evaluated by using the bootstrap method for the whole workflow, as described in the Methods section. Summary statistics (median, 95% confidence intervals and the relative standard errors) of the estimated values for all the model parameters are provided in Table 2. The relatively low standard errors and narrow 95% confidence intervals indicate that the workflow comprising the cluster analysis and the model fitting led to robust model parameter results. In other words, small changes in the dataset, conducted by resampling with replacement from the real data, led to similar estimates of the model parameters.

### 3.4. Bioequivalence Metrics Evaluation

**Figure 6:**
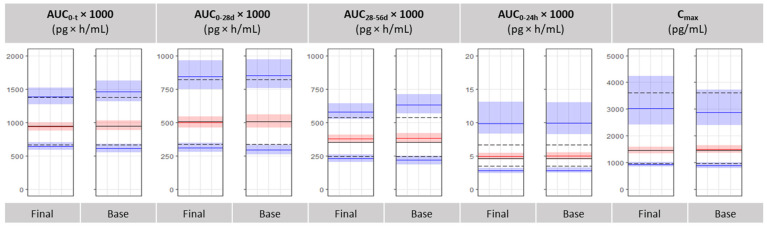
VPC plots for PK metrics in the base and final model. Black lines denote the median, 10th and 90th percentiles of the observations. The shaded areas and coloured lines represent the medians and 95% CI of the 1000 simulated datasets for the corresponding statistic measures of the observations. The five panels correspond respectively to the following PK parameters: AUC (0–t), AUC (0–28 days), AUC (28–56 days), AUC (0–24 h) and Cmax.

The performance of the base and final model to predict the pharmacokinetic parameters, which are usually used for the demonstration of bioequivalence, was tested. European Medicine Agency’s Committee for Medicinal Products for Human Use published a specific guidance for octreotide acetate depot powder to revise the requirements for bioequivalence demonstration as a waiver to a multiple-dose study, which is not practically feasible in healthy volunteers due to safety concerns, and not feasible in patients either due to the rareness of the disease. [16] A better characterization of the single dose PK study was required, comprising additional main and secondary PK parameters, among them the following: AUC (0–t), AUC (0–28 days), AUC (28–56 days), AUC (0–24 h) and Cmax. The VPCs of Figure 6, comparing model-predicted BE metrics to observed values calculated by Non-Compartmental Analysis, show that both population PK models describe well the aforementioned metrics in the study population, with the final model better predicting the 10th and 90th percentiles of Cmax and AUC (0–t), and partial AUCs. A model misspecification regarding the population distribution of the secondary parameter, partial AUC (0–24 h), is observed, as both the base and the final population PK model over-estimate the population variability of this variable.

## 4. Discussion

Limited information on the population pharmacokinetics of octreotide LAR has been formerly published. The aim of this work was to characterize octreotide LAR pharmacokinetics in humans, considering the empirical modelling of PK course in the individual level and the variability observed in the population. Octreotide LAR pharmacokinetics was modelled by a one-compartment model with linear elimination and an empirical release model, consisting of four phases, describing the absorption from the depot. A first-order absorption and three parallel transit processes with different delays described release. The variations of release patterns observed in the individual level are governed by the fraction Fi, the mean transit time MTT_i_ and the number of transit compartments N_i_, parameters. The rich PK dataset allowed the estimation of IIV for all the model parameters with low uncertainty. The final population PK model we developed, which incorporates sub-populations, describing well the octreotide PK course in both the individual and population level, and the PK metrics of AUC and Cmax.

Our work highlights that an important aspect of the PPK model development, especially for complex PK models, is the choice of the right parameterization for both the fixed and the random effects parameters [17]. Using the parameterization of the transit compartments model with mean transit time allowed the release empirical processes to be put in sequential order, which widened the space of local identifiability. Constraining the sum of individual fraction parameters (Σ*f_j_*_,*i*_) to one, and at the same time maintaining 0 ≤ *f_j_*_,*i*_ ≤ 1 was conducted with the use of the multivariate logistic-normal distribution. The aforementioned components of the model were crucial regarding the successful convergence of the estimation methods, reasonable computation time and precise estimates.

A PK study in rabbits has shown previously that octreotide LAR pharmacokinetics is characterized by three phases, a rapid initial burst followed by two delayed peaks. Empirical models, comprising an exponential, a semiempirical non-Fickian (power-law) and a delayed Weibull model were employed to describe the transient release of the drug from the microsphere surface, release from the polymeric matrix driven by diffusion and release phase due to polymer erosion, respectively. In general, the results of our PK analysis confirm the empirical PK model developed in the animal model. An additional phase was observed in our data, characterizing only one part of the cohort. The study design, including extensive sampling and the large number of subjects, limitations of the animal model and modelling assumptions may explain this discrepancy.

A novelty of the present study is the incorporation of the pre-processing step of the data with a method of unsupervised learning; a shape-respecting variation of k-means, which was used to explore patterns in the individual PK data. The idea of subjectively choosing individualized absorption models when multiple absorption profiles are evident in a population analysis was recently stated by Jaber et al. [18] in a different context. In the current work, we defined sub-populations that show different PK profiles before the population PK analysis, with limited human intervention and in a more rigorous manner. The clustering defined sub-populations were handled as categorical covariates and the model’s overall goodness-of-fit was evaluated. Covariates of size and age failed to explain the population variability of the parameters, which is responsible for the two evident phenotypes. For the model to reproduce realistic individual PK curves at the observed frequency, defining sub-populations was inevitable. This workflow may be an alternative when mixture modelling is not feasible, due to identifiability issues or inability of the estimation method to converge, as it was in our case. This “shape-respecting” clustering method may be valuable in identifying sub-populations in pharmacokinetic or pharmacodynamic, longitudinal data and is sensitive enough, even in cases of unbalanced occurrence of the sub-populations.

The lack of a typical PK course for octreotide LAR reveals the difficulties in predicting the PK of long-acting injectable products. The release rate may depend on many factors: formulation-dependent, such as drug load, microparticle size, system-dependent, such as the dynamics of pore formation, agglomeration and host-dependent, such as the local immune response and muscle tissue physiology. Our study confirms that octreotide LAR formulation succeeds to control the huge initial burst observed in other LAIs. To be specific, initial burst accounts for less than 1/100 of the total exposure, while only two subjects out of the 118 exhibited the Cmax over this phase. Octreotide LAR is the only PLGA-based LAI formulation made of branched PLGA (also called star-shaped PLGA), which contains glucose [3]. It is not known whether systems based on branched PLGA have different release properties compared with linear PLGA, while an analytical technique to characterize branched PLGA was not developed until recently [19]. Questions concerning the capability of in vitro experiments to explain the mechanisms of release for long-acting injectable products is, according to our view, reasonable. The population PK model we developed for octreotide LAR may be useful for the evaluation of hypotheses regarding the underlying pharmacokinetic mechanisms for these type of products, from a bottom-up point of view.

## 5. Conclusions

The population PK model developed describes the variable and complex pharmacokinetics of the long-acting injectable formulation, octreotide LAR. We proposed a workflow, showing that cluster analysis may be valuable in cases where sub-populations are present. The “right” level of granularity in modelling was chosen to provide insights on the release properties and good representation of exposure.

## Figures and Tables

**Table 1:** Demographic, clustering and non-compartmental analysis data.

Demographics	Median (Q1–Q3)
Subjects, n	118
Age, years	28 (23–37)
Height, cm	175 (170–178)
Weight, kg	75 (66–86)
BMI, kg/m^2^	24.75 (22.4–27.7)
**Clustering**	
Cluster 1, n	103
Cluster 2, n	15
**Non-Compartmental Analysis**	**Mean (±SD)**
AUC_0–t_ (pg × h/mL)	988.7 × 10^3^ (±327.9 × 10^3^)
Cluster 1	944.0 × 10^3^ (±284.0 × 10^3^)
Cluster 2	1295.2 × 10^3^ (±442.1 × 10^3^)
Cmax (pg/mL)	1891.1 (±1622.6)
Cluster 1	1433.3 (±497.4)
Cluster 2	5034.8 (±2840.6)

**Table 2:** Parameter estimates of the final model and the corresponding inter-individual variability. Relative standard errors and bootstrap confidence intervals are also provided.

Parameter	Estimate (RSE%)	Bootstrap			Workflow Bootstrap		
		Median	95% CI	RSE (%)	Median	95% CI	RSE (%)
k_a_	0.27 (2.2)	0.27	0.26–0.28	2	0.27	0.26–0.28	2
V	15.3 (7.7)	15.1	13.5–16.8	6	15.1	13.6–17.1	6
CL Cluster effect:	32.7 (5.8) −8.61 (34)	32.7 −9.32	31.0–34.4 −14.32 to −3.66	3 48	32.6 −9.24	30.8–34.8 −13.95 to −3.57	3 38
Y_F1_	−5.18 (1.8)	−5.19	−5.24 to −5.11	1	−5.19	−5.24 to −5.11	1
Y_F2_ Cluster effect:	−3.36 (7.9) 3.06 (33)	−3.35 3.01	−3.62 to −3.03 2.52–3.46	4 8	−3.34 3.02	−3.69 to −3.02 2.02–3.55	5 14
Y_F3_ Cluster effect:	−1.54 (2.8) −0.523 (26.8)	−1.54 −0.468	−1.64 to −1.42 −0.704 to −0.291	4 22	−1.55 −0.47	−1.65 to −1.44 −0.715–0.01	4 35
Y_MTT1_	−0.421 (21.8)	−0.41	−0.554 to −0.244	20	−0.41	−0.562 to −0.253	20
MTT2	181 (3.3)	180	166–191	5	179	167–191	4
MTT3	506 (3.8)	508	486–530	4	508	481–534	3
N1	3.42 (15)	3.43	2.67–4.07	10	3.44	2.80–4.10	9
N2	17.9 (6)	18.0	15.1–20.2	7	18.0	15.9–20.3	7
N3	5.08 (5)	5.00	4.57–5.62	5	5.03	4.58–5.57	5
Proportional Residual Error	0.143 (1.3)	0.143	0.128–0.155	5	0.14	0.127–0.156	5
Additive Residual Error	28.4 (3.7)	28.2	22.9–33.8	10	28.8	23.6–35.2	10
**Inter-Individual Variability**	**Estimate (RSE%) [Shrinkage %]**	**Median**	**95% CI**	**RSE (%)**	**Median**	**95% CI**	**RSE (%)**
IIV_V_	39.4 (13) [16.3]	39.9	33.4–46.5	17	39.7	32.1–46.1	13
IIV_CL_	28.2 (8) [1]	28.3	24.2–34.6	30	28.3	23.4–32.7	22
IIV_YF1_	28.9 (7) [3.4]	25.8	14.1–50.1	65	28.1	13.9–48.2	50
IIV_YF2_	128.8 (16) [4]	128.4	112.5–141.3	6	129.2	110.9–143.7	6
IIV_YF3_	20.5 (18) [30.3]	21.0	13.1–35.0	30	21.1	14.1–37.2	25
IIV_YMTT1_	60.1 (12) [12]	59.6	48.5–70.7	10	60.6	49.5–73.6	10
IIV_MTT2_	17.3 (20) [17.6]	18.2	14.4–26.3	68	18.7	14.3–28.8	50
IIV_MTT3_	20.2 (9) [1.7]	20.7	16.9–31.2	74	20.6	16.6–30.0	54
IIV_N1_	71.2 (10) [22]	70.2	36.0–101.5	25	69.2	36.0–106.1	23
IIV_N2_	26.2 (21) [31.2]	26.3	16.0–33.6	32	26.5	16.9–34.1	26
IIV_N3_	31.4 (12) [9.3]	29.7	24.0–41.5	14	31	24.2–42.2	14

RSE, relative standard error; CI, confidence interval; ka, absorption rate constant; CL, apparent clearance; V, apparent volume of distribution; Y_Fi_, normal variable associated with the fraction of the i_th_ transit process; MTT_i_, mean transit time of the i_th_ process; YMTT1, normal variable associated with MTT1; Ni, number of transit compartments for the i_th_ process.

## Data Availability

The data presented in this study are available on request from the corresponding author.

## Supplementary Materials

The following are available online at https://www.mdpi.com/article/10.3390/pharmaceutics13101578/s1, Figure S1: Observations vs individual predictions in the linear and the logarithmic scale. Figure S2: Goodness-of-fit plots for the evaluation of the final PPK model. Table S1: Parameter estimates of the base model and the corresponding inter-individual variability.
