# Supplementary material for: Population Pharmacokinetic Modelling of the Complex Release Kinetics of Octreotide LAR: Defining Sub-Populations by Cluster Analysis
Source: Pharmaceutics. 2021 Sep 28;13(10):1578. doi: 10.3390/pharmaceutics13101578 (PMC8537465; doi:10.3390/pharmaceutics13101578)
Supplement: Supplementary file 1 [file pharmaceutics-13-01578-s001.zip › pharmaceutics-1339504-supplementary.pdf]

pharmaceutics

Supplementary Materials:

Population Pharmacokinetic Modelling of the Complex Release Kinetics of Octreotide LAR: Defining Sub-Populations by Cluster Analysis

Iasonas Kapralos [1,2] and Aristides Dokoumetzidis [1,2,*]

[1] Laboratory of Biopharmaceutics-Pharmacokinetics, Department of Pharmacy, National and Kapodistrian University of Athens, Athens 15771, Greece; iasonaskap@gmail.com
[2] Athena Research and Innovation Center in Information, Communication and Knowledge Technologies, Athens 15125, Greece
* Correspondence: adokoum@pharm.uoa.gr

Table S1. Parameter estimates of the base model and the corresponding inter-individual variability. Relative standard errors and bootstrap confidence intervals are also provided.

Parameter	Population Mean		Inter-individual Variability		
	**Estimate**	**RSE%**	**Estimate**	**RSE%**	**Shrinkage%**
k_a	0.269	0	0 FIXED	-	-
V	14.9	6.1	40.4	13	16
CL	31	6.9	33.9	16	0.1
Y_{F1}	−5.19	0.8	18.9	12	8.7
Y_{F2}	−2.98	10	165.5	17	2.8
Y_{F3}	−1.58	2.8	24.6	14	23
Y_{MTT1}	-0.395	23.8	60.5	14	15.4
MTT2	180	4.3	22.2	20	15.6
MTT3	515	5.4	24.5	9	0.7
N1	3.31	14.4	72.7	10	22.2
N2	17.5	6.1	27.9	22	30.9
N3	5.12	4.5	29.4	11	11.1
Proportional Residual Error	0.147	1.3	-	-	-
Additive Residual Error	27.1	4.2	-	-	-

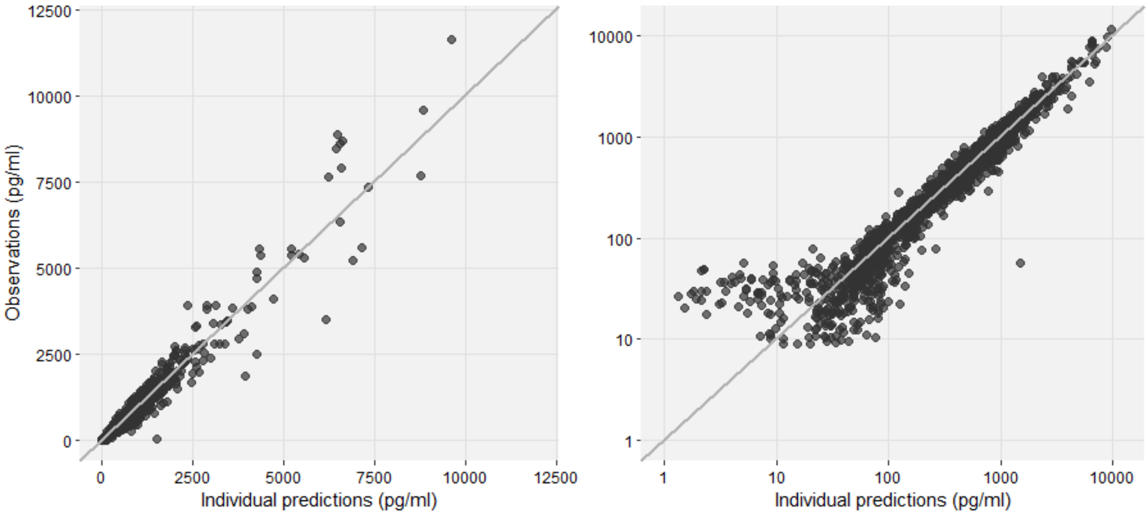

(a) **(b)**

Figure S1. Observations vs individual predictions in the linear (**a**) and the logarithmic (**b**) scale. A misspecification of the model is evident with the underprediction of the low concentrations, at the terminal slope of the PK curve. The empirical release model was not feasible to explain this data.

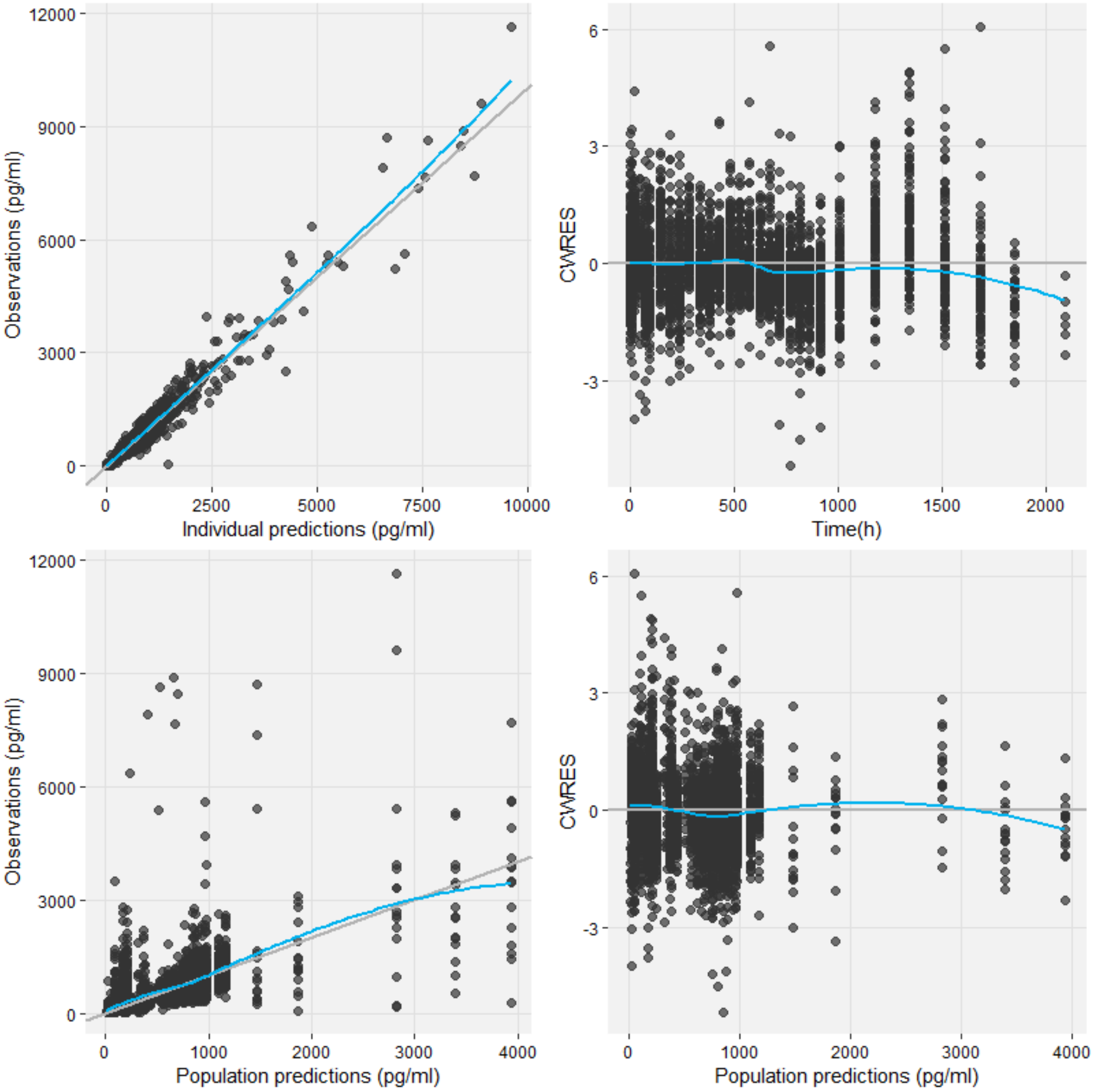

Figure S2. Goodness-of-fit plots for the evaluation of the final PPK model.
